# Abscopal Effects: Case Report and Emerging Opportunities

**DOI:** 10.7759/cureus.344

**Published:** 2015-10-07

**Authors:** Michael Lock, Ahmad Muinuddin, Walter I Kocha, Robert Dinniwell, George Rodrigues, David D'souza

**Affiliations:** 1 Department of Radiation Oncology, London Regional Cancer Program, London, Ontario, CA; Schulich School of Medicine & Dentistry, Western University, London, Ontario, CA.; 2 Oncology, Trillium Health Partners; 3 Oncology, London Health Sciences Centre; 4 Cancer Clinical Research Unit (CCRU), Princess Margaret Cancer Centre; 5 Department of Radiation Oncology, London Regional Cancer Program, London, Ontario, CA; Schulich School of Medicine & Dentistry, Western University, London, Ontario, CA; 6 Department of Radiation Oncology, London Regional Cancer Program, London, Ontario, CA; Schulich School of Medicine & Dentistry, Western University, London, Ontario, CA.

**Keywords:** abscopal effect, lung metastases, radiotherapy, liver cancer, spontaneous regression, immunomodulation, immune checkpoint inhibitors, sbrt, lung tumors

## Abstract

The abscopal effect is a phenomenon observed in the treatment of metastatic cancer where localized irradiation of a particular tumor site causes a response in a site distant to the irradiated volume. The mechanisms of the abscopal effect are speculated to be of several origins, including distant effects on p53, elaboration of inflammatory agents including cytokines, and, most recently, secondary to immune mechanisms. In this case report, we present a rare report of a patient with hepatocellular carcinoma with lung metastases who, after receiving radiation treatment to the liver, had a treatment response in the liver and a complete response in the lung. Recent advances in the understanding of the primary role of immune-modulated cytotoxicity, especially with the success of immune checkpoint inhibitors, have the potential to turn the abscopal effect from a rare phenomenon into a tool to guide antineoplastic therapy and provide a new line of research.

## Introduction

Hepatocellular carcinoma (HCC) is the sixth most common malignancy worldwide, and the third most common cause of death from cancer [1]. The abscopal effect is a phenomenon rarely observed in the treatment of metastatic cancer where localized irradiation of a particular tumor site causes a response in a site distant to the irradiated area. Here, we describe a case report of a gentleman with HCC and lung metastases who, after receiving only focal radiation treatment to the liver, had complete and sustained radiological regression of pulmonary metastases.

The London Health Sciences Centre Research Ethics Board approved this study (approval #16487E). Patient information was collected and released under an ethics approved prospective database.

## Case presentation

**Figure 1:**
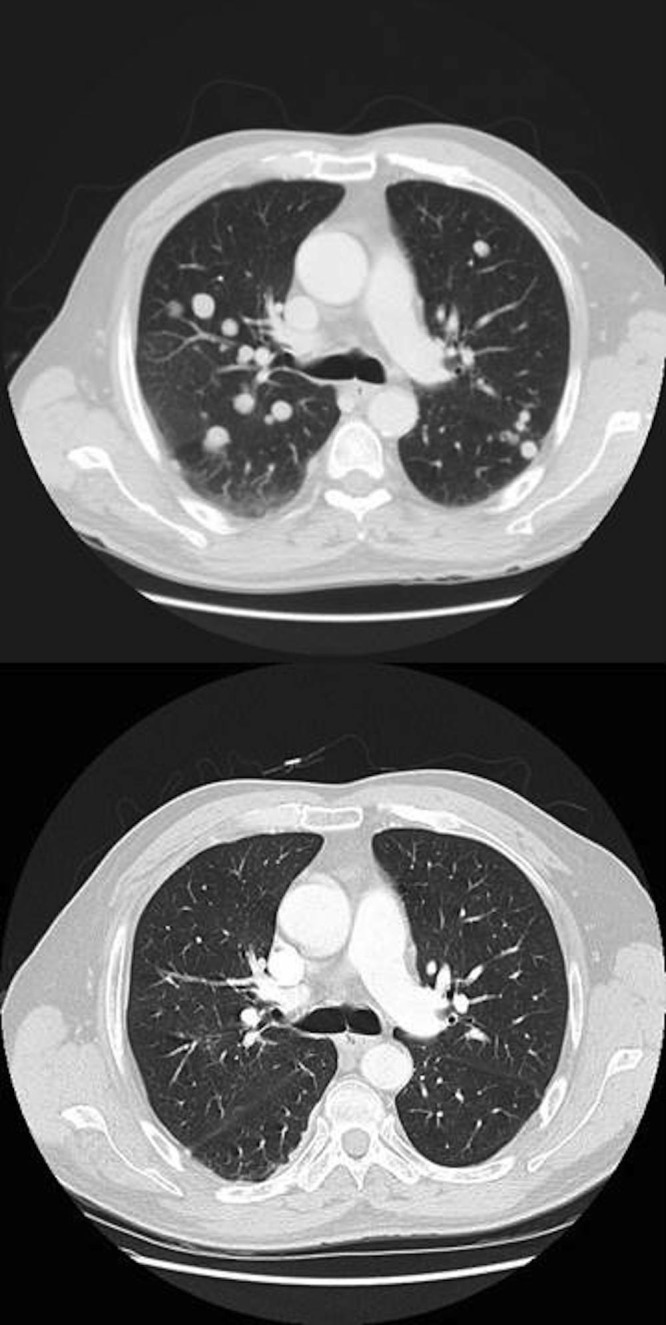
The axial CT scans of the thorax before and after radiation of the liver. Figure 1A (top) The February 2010 axial CT thorax demonstrates multiple metastases before radiation . Figure 1B (bottom) The August 2010 axial CT thorax demonstrates resolution of metastases five months after radiation .

In early December 2009, a 71-year-old man presented to the Emergency Department with dyspnea as well as right-sided chest wall and upper quadrant abdominal pain. He was diagnosed with pulmonary embolus and started on anticoagulation. His imaging investigations revealed multiple coalesced masses in the liver with the largest measuring 6 cm x 9 cm x 9 cm in the right lobe of the liver. Multiple lesions were also noted in the lung with bilateral pleural plaques consistent with asbestos exposure (Figure 1A). Alpha-fetoprotein (AFP) was significantly elevated at 11 460 µg/L (normal less than 5). In late December, a liver biopsy was performed and pathology was diagnostic for primary HCC. He was diagnosed with Stage IV T3N0M1 disease. His past medical history was only significant for hypertension and Type 2 diabetes. He was an ex-smoker and quit in 1968. He was a retired boilermaker with asbestos exposure.

In late January 2010, he met with a liver surgeon. A repeat CT scan of the thorax and abdomen in early February revealed the liver lesions had increased in size with the primary lesion now measuring greater than 14 cm. Furthermore, there was evidence of worsening metastatic disease in the lungs with innumerable lesions between 0.5 and 2 cm in size. These were non-calcified and non-cavitary lesions. With this rapid doubling time, there was some debate within the Multidisciplinary Tumor Board about the role of medical treatment with sorafenib. The patient was ECOG 1 and had only mild hepatomegaly. He had no jaundice or stigmata of liver disease but was experiencing significant fatigue. Liver enzymes were minimally elevated with ALT 31 IU/L (normal less than 40), AST 73  IU/L (normal less than 41), ALP 95  IU/L (normal less than 129), and total bilirubin 8.1 µmol/L (normal less than 17). His albumin was 35 g/L and INR was normal. His Child-Pugh Score was A6.  However, the combination of his hypertension and anticoagulation raised the concern of increased bleeding risk and potential difficulty controlling hypertension while on sorafenib. Therefore, in conjunction with the patient, it was decided to go ahead with radiotherapy alone.   

**Figure 2:**
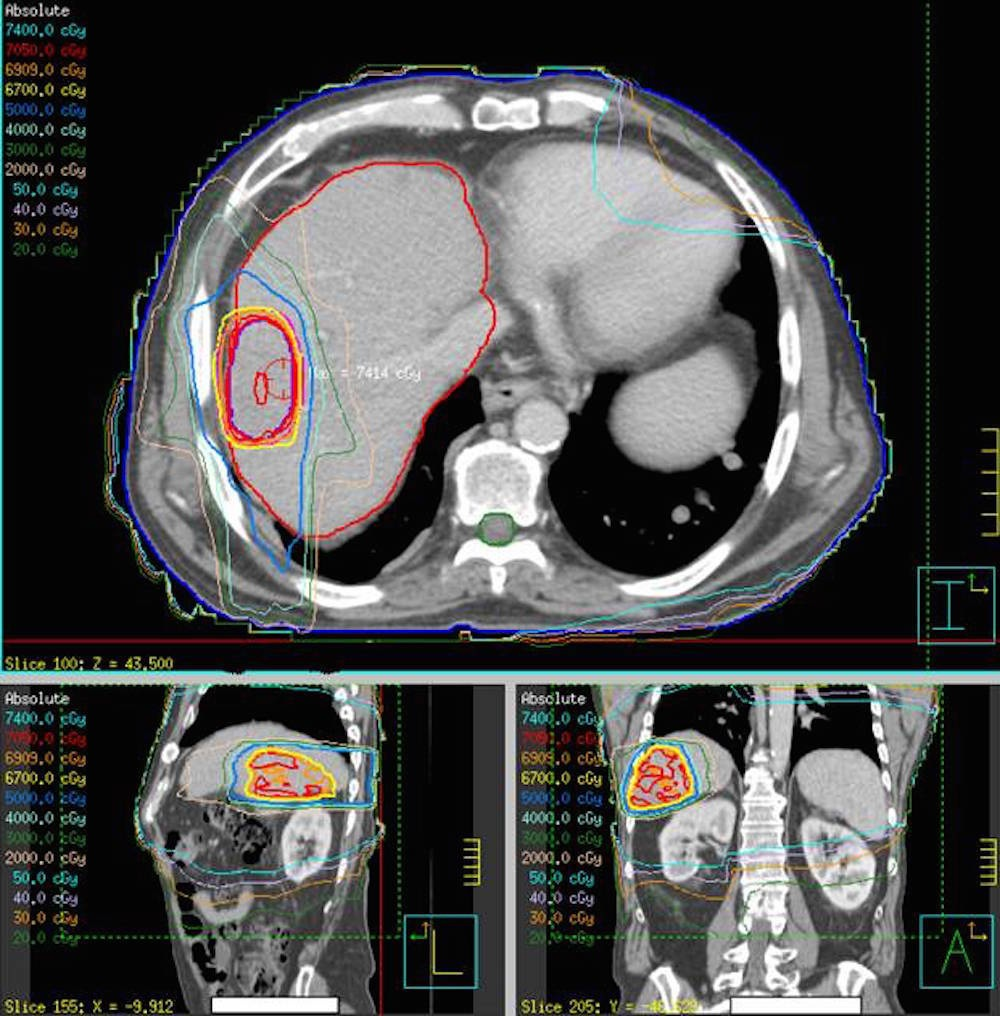
Radiation dosimetry plan for liver treatment The patient underwent 70 Gy treatment in 15 fractions to the liver which was completed in April 2010.

The patient underwent 70 Gy treatment in 15 fractions to the liver, which was completed in April 2010 (Figure 2). A follow-up CT abdomen in June 2010 showed the largest liver lesion had now shrunk from over 14 cm to 3 cm. The patient was clinically stable and asymptomatic. Biochemically, the AFP had decreased to 196.8 µg/L. In August 2010, the CT thorax and abdomen demonstrated stable disease in the liver. Furthermore, there was a dramatic complete resolution of the numerous pulmonary metastases with no new nodules or masses seen (Figure 1B). The patient received no other treatments up to this assessment point (August 2010), including no systemic treatments.

From August 2010 till March 2012, the man enjoyed stable disease with normal liver laboratory results (including an AFP of 3.1) and no imaging evidence of disease recurrence. In March of 2012, a small recurrent nodule was found in the right liver and he underwent hepatic artery embolization with doxorubicin and cisplatin in May of 2012. This resulted in a complete response at that location.

## Discussion

In 1953, Mole described the phenomena of abscopal effect as an “action at a distance from the irradiated volume, but within the same organism” [2]. It can result in a tumor in a non-irradiated area being spontaneously reduced. Since Mole’s original account, many cases of the abscopal effect have been described in multiple tumor sites, including lymphoma and melanoma [3-4]. Though there are some reports of abscopal effect in hepatocellular carcinoma, these primarily report regression of hepatocellular carcinoma itself due to radiation treatment delivered at a metastatic site or with systemic treatment [5-10]. To our knowledge, ours is the first case report of the abscopal effect with focal liver radiation causing regression of distant lung metastasis in a systemic treatment naïve patient with HCC.  

One interesting report in the literature describes a man with HCC who had a mediastinal metastasis and a pulmonary mass in the right lower lobe. After receiving focused mediastinal radiation, the abscopal effect was observed with regression of the pulmonary mass, which was not in the irradiated field [10]. However, in this case, the patient was first treated with a transcatheter arterial embolization, and hence, it is unclear if this treatment may have contributed to the response. This is different from our case, in which the patient was naïve to any treatment and received radiation treatment to the liver only, with subsequent regression of distant lung metastases. A limitation of our case report is that there was no pathologic confirmation for the metastatic disease in the lungs. However, the CT images (Figure 1A) reported by the radiologists were convincing and consistent with the common “cannon ball” lesions seen in lung metastasis. 

The mechanism of the abscopal effect is still not well understood, although major mechanistic categories have been proposed and opportunities for exploitation reviewed [11-37]. These include involvement of the immune system, cytokines, tumor growth inhibition, and tumoricidal effects [16, 25, 38-39]. One proposed mechanism is that the irradiation of one tumor site results in the release of circulating tumor antigens and inflammatory factors that then mediate an augmented immune response against non-irradiated malignant lesions that express the same tumor antigens [40-41]. A French study [42] investigated the radiation-induced inflammatory response in mice after total abdominal or total-body irradiation. A systemic inflammatory reaction was found after abdominal and total-body irradiation in these mice, concomitant with increased cytokine and chemokine production in the jejunum and also in the lungs [42].

More recently, the observation of unexpected abscopal response in patients receiving radiation during immunotherapy has attracted much interest. Many of these mechanisms have explicit support from animal studies [40, 43-50, 65]. There is considerable insight into the mechanisms of synergistic activity between radiation immunogenic cell death and other biological effects. There appears to be a promotion of T cell recruitment and function associated with irradiation [51-52]. Roles for TGF-β [53], involvement of p53 [54-57], and local tumor damage exposing previously hidden antigens have been implicated [40-41]. Upregulation of cytokines has also been demonstrated [38-39]. Immune checkpoint research has shown remarkable clinical results and may play a principle role when combined with radiation [15, 58-59]. CTLA-4, an immune checkpoint protein, has been shown to downregulate the immune system. In landmark work by Dewan, anti-CTLA-4 antibodies were assessed with and without radiation [59]. Dewan demonstrated that immune checkpoint inhibition alone or radiation alone did not result in an abscopal effect. However, a combination of fractionated radiation and anti-CTLA-4 results in an abscopal effect [59]. Inhibition of the secondary tumor was proportional to CD8+ T cells showing tumor specific IFN-gamma production. This synergy with immune checkpoint inhibitors can result in the net effect on antibodies targeting inhibitory receptors on T cells [4, 58-64]. In a 2015 review of 13 preclinical and 24 clinical papers, Reynders, et al. found that time to a documented abscopal response was within 24 months (median of 5 months). The response was maintained for 3-39 months (median of 13 months) [65]. 

## Conclusions

We have reported a case of abscopal effect with focal radiation therapy to the liver causing regression of distant pulmonary metastasis in HCC. There is increasing evidence supporting the mechanisms of the abscopal effect as an immune-mediated process initiated by high-dose radiation. The recent elaboration and utility of immune checkpoint inhibitors in clinical practice provides significant optimism of a role of radiotherapy in combination with these therapies. This is a promising area of research and therapeutic development, which could lead us to take advantage of these phenomena in the treatment of cancer.
